# Data-Driven Identification and Analysis of the Glass Transition in Polymer Melts

**DOI:** 10.1021/acsmacrolett.2c00749

**Published:** 2023-05-11

**Authors:** Atreyee Banerjee, Hsiao-Ping Hsu, Kurt Kremer, Oleksandra Kukharenko

**Affiliations:** Theory Department, Max Planck Institute for Polymer Research, Ackermannweg 10, 55128 Mainz, Germany

## Abstract

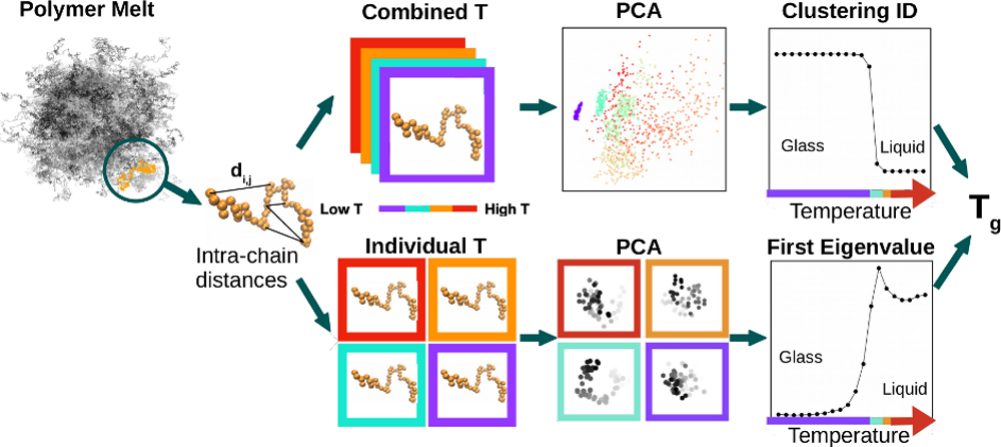

Understanding the nature of glass transition, as well as the precise estimation of the glass transition temperature for polymeric materials, remains open questions in both experimental and theoretical polymer sciences. We propose a data-driven approach, which utilizes the high-resolution details accessible through the molecular dynamics simulation and considers the structural information on individual chains. It clearly identifies the glass transition temperature of polymer melts of weakly semiflexible chains. By combining principal component analysis and clustering, we identify the glass transition temperature in the asymptotic limit even from relatively short time trajectories, which just reach into the Rouse-like monomer displacement regime. We demonstrate that fluctuations captured by the principal component analysis reflect the change in a chain’s behavior: from conformational rearrangement above to small fluctuations below the glass transition temperature. Our approach is straightforward to apply and should be applicable to other polymeric glass-forming liquids.

Polymer materials in applications are often in the glassy state. Upon cooling of a rubbery liquid polymer, dynamic properties such as viscosity or relaxation time increase drastically near the glass transition temperature (*T*_g_) in a super-Arrhenius fashion^1−4^ without any remarkable change in structural properties.^3^ Despite enormous experimental and theoretical efforts,^5−10^ the nature of glass transition as well as the question of a precisely defined *T*_g_ still remain unclear.^4,11−14^ In computer simulations, *T*_g_ is often calculated from characteristic macroscopic properties, e.g., changes in the specific volume, density, or energy.^14−16^ The increase in viscosity, equivalent to the terminal relaxation times, is commonly fitted to a Vogel–Fulcher–Tamann behavior that predicts a divergence at *T*_VFT_,^17^ typically about 50° below the calorimetric *T*_g_.^18^ However, the precise value of the observed *T*_g_ depends on the cooling rate and fitting procedures, which can lead to some ambiguities in comparison with experimental values,^19,20^ unlike a sharp and distinct change in physical properties. Thus, reliable predictions of *T*_g_ are indeed challenging.^12,13,21,22^

Attempts to link *T*_g_ with the molecular structure of polymeric materials draw more attention. Recent studies predict *T*_g_ by quantifying the changes in specific dihedral angles and transitions between states defined by those angles^13,14^ or by using averaged intrachain properties.^23^ A possibility to specify the structural properties of the glassy systems, which can reflect changes in *T*_g_, is attractive, but it remains challenging and system specific. Machine learning (ML) methods hold great promise to automatize the determination of structural descriptors from molecular simulation data. Recently, the application of ML to nonpolymeric supercooled model liquids allowed to understand the connection between characteristic local structures and the slowing down of dynamical properties.^24−29^ For polymer chains in a melt, the intrachain properties associated with the chain connectivity and flexibility also play an important role in determining *T*_g_. However, the application of ML methods to determine structural changes during the glass transition in polymer chains is limited.^13,30,31^

In this Letter, we use unsupervised data-driven methods to identify the glass transition of polymer melts of weakly entangled polymer chains only by employing information about conformational fluctuations at different temperatures. We first analyze the combined data from different temperatures using principal component analysis (PCA),^32^ followed by clustering and determine a clear signature of glass transition. Considering the simulation data within a finite observation time window up into the Rouse-like regime, our approach allows a very solid extrapolation to infinite times to predict *T*_g_. We then also employ the data-driven methods on individual temperature data separately. The nonmonotonic variation of the magnitudes of leading eigenvalues and the participation ratio derived from PCA captures the signature of the glass transition. It also reflects a change in the nature of the fluctuations in the system. We apply these approaches to the simulation data of a coarse-grained polymer model^33^ and compare estimates of *T*_g_ obtained from classical fitting of macroscopic properties with the new method. The proposed method has the following advantages: (a) our approach is based on high-resolution microscopic details instead of average macroscopic properties, (b) it does not rely on the fitting protocols, and (c) our analysis focuses on the information about structural fluctuations at the level of individual chains to predict *T*_g_ from very moderate simulation trajectories.

In ref (33), Hsu and Kremer developed a new variant of the bead–spring model^34,35^ for studying the glass transition of polymer melts.^36,37^ Molecular dynamics simulations of a bulk polymer melt containing *n*_c_ = 2000 semiflexible polymer chains of chain length *n*_*m*_ = 50 monomers and a Kuhn length ≈ 2.66σ^38^ were first performed in the NPT ensemble at *P* ≈ 0ϵ/σ^3^ and constant *T* following a standard stepwise cooling protocol (20 temperatures from 1.0 to 0.05 ϵ/*k*_B_), choosing a fast fixed cooling rate of Γ = 8.3 × 10^–7^ϵ/(*k*_B_τ) (see Supporting Information (SI), Sec. S-I for details). The Rouse time is , and the entanglement time is  with the characteristic relaxation time τ_0_ ≈ 2.89τ estimated at *T* = 1.0ϵ/*k*_B_, and the entanglement length *N*_e_ = 28 monomers.^38^ Here τ_0_ is the upper limit of time that a monomer can move freely. After the step cooling, subsequent NVT runs up to 3 × 10^4^τ were performed at each *T* to investigate the monomer mobility characterized by the mean square displacement *g*_1_(*t*) (for details, see SI, Sec. S-I, Figure S1). In this Letter, we mainly use simulation trajectories from NVT runs stored every 200τ in the time window between 200τ and 3 × 10^4^τ (gray area in Figure S1, SI), resulting in 150 time frames per temperature.

The first estimate of the glass transition temperature at *T*_g_ ≈ 0.64ϵ/*k*_B_ using a conventional fitting procedure was determined from the volume change (Figure 1a, inset).^33^ We here adapt another standard approach to estimate *T*_g_ by performing a hyperbolic fit^39^ on the temperature-dependent density of polymer melt for 0.1 ≤ *k*_B_*T*/ϵ ≤ 1.0, , where *c*, *T*_0_, *a*, *b*, and *f* are fitting parameters. *T*_g_ is either defined by *T*_g_ = *T*_0_ or the intersection point of the two tangents drawn at the high and low temperatures. Both give an identical, more precise, estimate of *T*_g_ = 0.660(4)ϵ/*k*_B_, as shown in Figure 1a, and are used as reference values for evaluating the data-driven approach presented below. Note that *T*_g_ obtained from the simulation data depends on the cooling rate. We propose here an alternative data-driven approach to gain insight into the glass transition with a minimum a priori knowledge about the system and user input.

**Figure 1:**
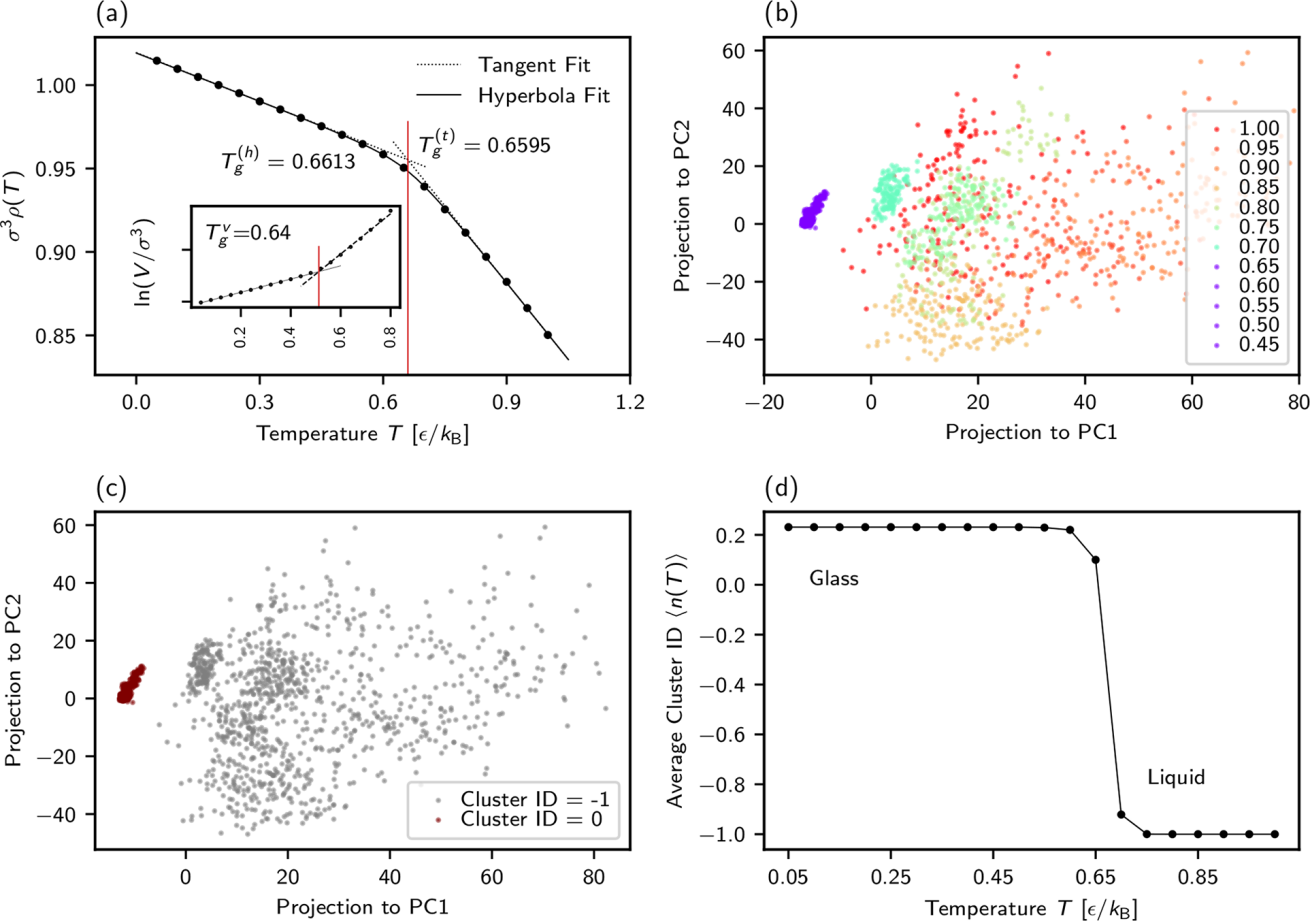
(a) Conventional methods of estimating the glass transition temperature *T*_g_: density (ρ(*T*)) and logarithm of volume (ln(*V*/σ^3^)) (in the inset) plotted versus *T*. Estimates of *T*_g_ via the two tangent (t) fits (dotted lines) at high and low *T*, hyperbola (h) fit (curve), and two linear fits (dashed lines in the inset) are indicated by vertical lines. (Data are taken from ref (33).). (b–d) Data-driven determination of *T*_g_. (b) Projections of concatenated data from all *T* for a single chain over multiple time frames in the two first leading principal components (PCs). Each point in the plot corresponds to one chain’s conformation at a given temperature at each time. Projections for *T* > *T*_g_ are colored varying from red to green while they are in purple for *T* < *T*_g_ (data shown for *T* ≥ 0.45 for clarity). Note that the axis values in the PCA embedding do not correspond to a directly measurable physical quantity, rather could be viewed as a weighted linear combination of scaled input distances. (c) DBSCAN of the PCA projection. The same projection as (b), but it is colored with DBSCAN cluster indices (ID) instead of temperature. DBSCAN assigns the high-temperature liquid state as noise (cluster ID = −1) and the low-temperature glassy state as a cluster (cluster ID = 0). (d) Average cluster ID over all chains versus *T*. The separation between the liquid and glass state becomes sharper if we use median instead of mean (see SI, Figure S6b).

The analysis workflow consists of two different, but related, methods (a sketch is given in SI, Sec. S-III). Both identify the same *T*_g_, but treat the data differently (using combined information from all 20 temperatures or individual information from each temperature). To identify changes in the studied systems, we first define possible descriptors: sets of all pairwise internal distances for a single chain. They are well suited to describe conformational fluctuations of individual polymer chains. Then we apply PCA^32^ to the high-dimensional descriptor space. PCA has been successfully used to characterize the phase transition in conserved Ising spin systems.^40,41^ The method relies on purely structural information without any a priory knowledge of dynamical correlations. A *M* × *L* real matrix **X**_*c*_ with elements , 1 ≤ *m* ≤ *M*, 1 ≤ *l* ≤ *L* is used to represent data for a single chain *c*. Here *c* = 1, ..., *n*_*c*_ is a chain index, *L* is the number of descriptors (e.g., the intrachain distances between any two monomers in a single chain of *n*_*m*_ = 50 monomers: *L* = *n*_*m*_ × (*n*_*m*_ – 1)/2 = 1225), and *M* is the number of observations (i.e., *M* = 150 (time frames) × 20 (temperatures) = 3000 for Method I, and *M* = 150 (time frames) × 1 (temperature) = 150 for Method II). **X**_*c*_ is standardized column-wise, i.e., each element  is converted to , where  is the mean value for each column *l*, and  is its corresponding standard deviation for chain *c* such that the rescaled columns  have a mean value of 0 and a variance of 1. PCA is done individually for each chain by first calculating the covariance matrix , where , 1 ≤ *j*, *k* ≤ *L* are elements of **C**_*c*_, and ,  are the standardized descriptors. Then the eigenvalues λ_*c*,*i*_ and the corresponding eigenvectors **v**_*c*,*i*_ of the matrix *C*_*c*_, for *i* = 1, 2, 3, ..., min(*L*, *M*) are calculated and sorted in decreasing order of λ_*c*,*i*_. The original data set **X**_*c*_ is converted to  by projecting **X**_*c*_ to the new orthogonal basis formed by *P*-leading eigenvectors **v**_*c*,*k*_, where elements of  are , *k* = 1, ..., *P*, and *P* ≤ min(*L*, *M*) is the reduced number of dimensions (*P* = 4 in this work).

Due to the correlated motions of neighboring monomers, the intrachain distance space can be reduced by skipping some distances. We discuss this in more detail in SI, Sec. S-IX. All results are similar in nature after reducing the input feature space, and the asymptotic estimate of the glass transition temperature is reported considering every fifth monomer in a chain.

*Method I*: We perform PCA on a randomly selected single chain using the internal distances over time concatenated for all temperatures. In this way we construct the new basis formed by eigenvectors **v**_*c*,*i*_ containing information about fluctuations of internal distances at all temperatures. The internal distances of the chain at each simulation snapshot and temperature are projected independently on this new basis. Thus, projections in the new PCA space can be viewed as linear combinations of input distances. Already in the two-dimensional projection one could clearly differentiate between two states (Figure 1b), which occur roughly around the glass transition temperature *T*_g_ ≈ 0.65ϵ/*k*_B_ (Figure 1a). The scatter of the PCA projection qualitatively changes at and below 0.65ϵ/*k*_B_, indicating the onset of a different state.

To quantify the separation between the liquid and glassy state, we perform such a PCA for each chain separately, followed by clustering. Clustering groups the chains’ conformations at each simulation snapshot and temperature based on similarities in their conformational fluctuations reflected as closeness in the PCA projection space. Thus, each chain conformation is assigned an index corresponding to the group it belongs to. Such an index is called a cluster index (ID). We used density-based spatial clustering of applications with noise (DBSCAN)^42^ for each projection in a four dimensional space of leading principal components (PCs). The cluster ID *n*_*i*_ for a single chain at each time frame is always an integer, i.e., *n*_*i*_ ∈ {−1, 0, 1, 2, ..., *n*_cluster_ – 1} for *i* = 1, 2, ..., *n*_*c*_*M*, where the number of chain *n*_*c*_ = 2000, the number of frames *M* = 150 at each *T*, *n*_cluster_ is a number of clusters found by DBSCAN (max(*n*_cluster_) = 3 in this work). *n*_*i*_ = −1 corresponds to the noise, while *n*_*i*_ ≥ 0 corresponds to the clusters found in the four-dimensional PCA projections using DBSCAN.^42^ The details of clustering, the rationale for choosing four dimensions in PC space, the goodness of clustering are given in the SI (Sec. S-IV, S-V). DBSCAN determines the high-temperature states as sparse or “noise” (and assigns them with cluster ID = −1) and the low-temperature glassy state as a cluster(s) (Cluster IDs ≥ 0), see, e.g., Figure 1b,c. Then, we repeat this clustering on each chain present in the system (2000 chains) to confirm that the separation between liquid and glassy state is consistent for all chains in the melt. To obtain a general estimate of the temperature at which this separation occurs, we calculate the average cluster ID ⟨*n*(*T*)⟩. At each temperature *T*, ⟨*n*(*T*)⟩ is given by , where *P*(*n*_*i*_, *T*) is the probability distribution of cluster IDs for all *n*_*c*_ chains over *M* frames at each *T*. In Figure 1d ⟨*n*(*T*)⟩ shows a sharp transition around *T* = 0.65ϵ/*k*_B_.

The glass transition is often viewed as the process of falling out of equilibrium during cooling at a given rate or as the onset of ergodicity breaking. Above *T*_g_ all states are accessible to the system, while below *T*_g_ the system is arrested. Therefore, we expect the dissimilarity between low and high temperature regimes at or around *T*_g_, giving rise to the sharp transition in the average cluster indices. Our result shows a signature of dynamic ergodicity breaking (Figure S1) indicated by a dramatic increase in the equilibration time (see the VFT-plot in ref (33)) for each chain at the same temperature; we report that as *T*_g_. A similar signature of ergodicity breaking has been reported recently using Jensen–Shannon divergence metric for homopolymers.^13^

To extrapolate obtained results to long time limits where the polymer chains are supposed to reach the diffusive regime, we repeated the analysis above for 16 observation time windows Δ*t* ranging from 1000τ to 3 × 10^4^τ. For each Δ*t*, we have used Δ*t*/*t*_lag_ consecutive frames with *t*_lag_ = 200τ. We see that the transition from the liquid to the glassy state becomes sharper with the increase of the observation time window (Figure 2). To quantify that, we interpolate the data by a hyperbolic tangent function *g*(*T*) = *C*(Δ*t*)(1 – tanh(*sT* – *d*))/2 – 1, where *s* and *d* are the fitting parameters, *C*(Δ*t*) is the gap between the two states at *T* ≫ *T*_g_ and *T* ≪ *T*_g_, respectively. The inflection point of *g*(*T*) gives the estimate of *T*_g_(Δ*t*), depending on Δ*t*. The behavior of average cluster ID vs *T*, as given in Figure 2, is similar to a typical behavior of magnetization vs *T* for a finite-size 2D Ising model^43^ and requires further investigation considering the existing discussion in the literature.^11^ The finite-size (time) effect is often considered for analyzing data obtained from simulations of finite system sizes or limited computing times. Taking into account this finite-time effect, we plot the estimates of *T*_g_(Δ*t*) versus 1/Δ*t* in the inset of Figure 2. We find a remarkable linear dependency, which allows for extrapolation to Δ*t* → *∞* and obtain *T*_g_ ≈ 0.6680ϵ/*k*_B_ as a best asymptotic estimate of *T*_g_. This is in excellent agreement with the classical analysis of the temperature-dependent density (Figure 1a).

**Figure 2:**
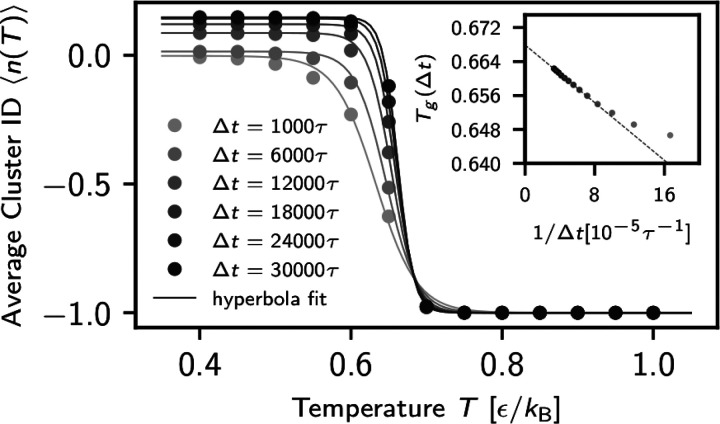
Average cluster ID ⟨*n*(*T*)⟩ for different selected observation time windows Δ*t*, as indicated. The curves give the best hyperbolic fit *g*(*T*) going through the data. The inflection point of *g*(*T*) shown in the inset gives the estimate of *T*_g_(Δ*t*) at each Δ*t*. Extrapolating to Δ*t* → *∞*, we obtain *T*_g_ ≈ 0.6680ϵ/*k*_B_.

In order to interpret the obtained projections to the leading PCs, we calculate the correlation between the internal distances and the corresponding projection to PCs (see SI, Sec. S-VII). The mostly correlated distances vary with different chains, with no clear signature of any characteristic distance. Due to standardization of the distances (i.e., see **X**_*c*_ definition for details), PCA accounts for relative changes in the distances rather than the absolute displacement values. As a result, the projections to leading PCs are not dominated by only large distances. However, they are related to physically motivated measures such as *R*_g_, *R*_e_ (other physical properties can be also compared) (SI, Figure S8d).

Note that we performed PCA on a single chain, followed by taking an average over all chains in the system. Performing PCA on 2000 chains combined, we only observe the same Gaussian-like distribution within fluctuations, stemming from different chains, which is essentially independent of the temperatures (see SI, Figure S7).

*Method II*: In the following, we change our approach and perform PCA for individual chains, but at different temperatures independently. In this way the new basis formed by eigenvectors **v**_*c*,*i*_(*T*) differ for each temperature (see SI, Sec. S-IX, showing examples of first eigenvectors for Methods I and II) and no information on individual chain conformations from other temperatures is accessible to Method II. The resulting projections are shown in SI, Figure S9. Notably, for the majority of chains in the melt, we could observe the change from a completely random distribution of points in the projection to more “clustered” with the decrease of *T*. This behavior can be quantified by the magnitude of the eigenvalues of PCA. In general, this magnitude is not a uniform value for independently projected data, but in our case all distances are standardized. Thus, we could average over the first eigenvalue for all projections (see Figure 3a), which shows a (weak) maximum close to *T*_g_. This suggests that above *T*_g_ large scale fluctuations dominate, while below *T*_g_ fluctuations are dominated by many contributions from different, but short length scales. As a more general criterion, we use the participation ratio (PR) defined at each temperature over 150 frames as , where λ_*c*,*i*_ are eigenvalues sorted in the descending order (see Figure 3b). PR reflects decay rate of eigenvalues: the steeper is the change the smaller PR will be, if all λ_*c*,*i*_ are equal then *PR* = *k*. A typical spectrum of eigenvalues λ_*c*,*i*_ with different decay rates are plotted in SI, Figure S4c. The leading *k* = 25 eigenvalues from min(*L*, *M*) eigenvalues are counted to preserve at least 80% data fluctuations in PCs. Results are averaged over all chains, deviations are shown as error bars. The increase in magnitude of the first eigenvalue (or the decrease in PR) on approaching *T*_g_ can be related to an appearance of state separation in the system and change in a local structure as some recent studies suggest.^13,14^ We argue that a prominent change in the monotonic behavior of PR (or the first eigenvalue) is connected with a change in the nature of the fluctuations in the system: from local configurational rearrangements (the rearrangement of parts of chain conformations) above *T*_g_ to only localized fluctuations along the chain below *T*_g_ (similar to observations in metallic glasses^44^). As a result, more dimensions are needed to describe the random motion below *T*_g_. To test the hypothesis about local structural changes above *T*_g_, we perform the same analysis on simulation trajectories within a relatively short time window between 0.2τ and 20τ (blue area in Figure S1). Results are shown in the inset in Figure 3. We no longer see the nonmonotonic signature around *T*_g_ since chains remain in their initial conformations within 1σ fluctuation in such a small time window. Projections of short-time data from individual temperatures are given in SI, Figure S10.

**Figure 3:**
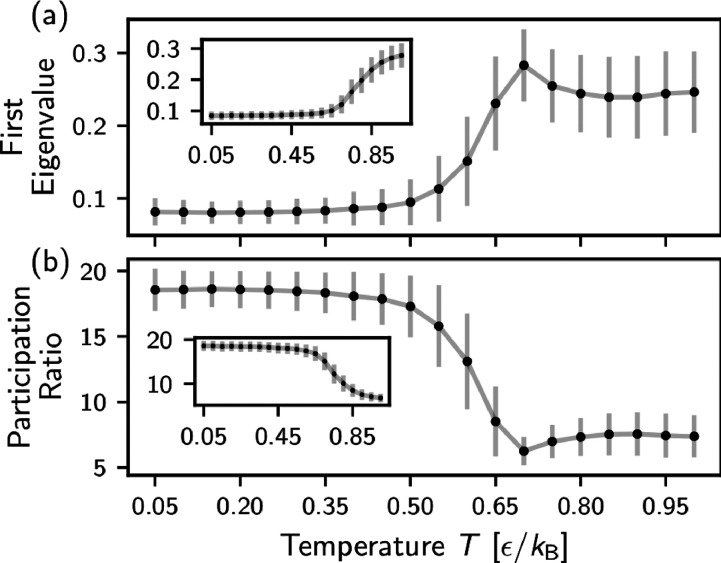
Analysis of each temperature independently. Mean values including deviations of the magnitude of first eigenvalues (a) and the participation ratio (b). Data taken from the time window between 200τ and 3 × 10^4^τ (gray area in Figure S1). The results for a shorter time up to 20τ (blue area in Figure S1) are shown in the insets.

In general, with method II, one can perform PCA on simulation trajectories at each temperature and monitor the eigenvalues and PR. Once we observe the nonmonotonic change in both quantities around *T*_g_, further simulations at lower temperatures are not required to localize *T*_g_.

In summary, we propose a new approach for determining the glass transition temperature from molecular dynamics simulation data with a fixed stepwise cooling protocol. The proposed data-driven protocol requires minimum input parameters and defines *T*_g_ in a robust and transferable fashion. Our analysis focuses on the information about structural fluctuations at the level of individual chains to identify the glass transition temperature and predict *T*_g_ for infinite simulation time from moderate simulation trajectories. We hypothesize that the relative distance fluctuations measured by the PCA may be directly correlated with the configurational entropy in the space of a single chain.^30^ The method can be applied to a wide range of systems with microscopic/atomistic information. The generality of our approach could be tested with different dimensionality reduction and clustering methods. Further work in this direction is in progress.

## Supporting Information Available

Simulation details (S-I), static properties of polymer melts (S-II), additional information on the data-driven approach (S-III), variance explained ratio of pca projections (S-IV), detailed description of clustering (S-V), principal component analysis on combined chains (S-VI), interpretation of leading principal components (S-VII), projections of a chain after performing PCA independently at each temperature (S-VIII), and results with reduced number of descriptors (S-IX) (PDF)
