# Supplementary material for: Data-Driven Identification and Analysis of the Glass Transition in Polymer Melts
Source: ACS Macro Lett. 2023 May 11;12(6):679–84. doi: 10.1021/acsmacrolett.2c00749 (PMC10286554; doi:10.1021/acsmacrolett.2c00749)
Supplement: Supplementary file 1 — mz2c00749_si_001.pdf [file mz2c00749_si_001.pdf]

Supporting Information to "Data-driven identification and analysis of the glass transition in polymer melts"

Atreyee Banerjee, Hsiao-Ping Hsu, Kurt Kremer, and Oleksandra Kukharenko

Max Planck Institute for Polymer Research,

Ackermannweg 10, 55128 Mainz, Germany

S-I. SIMULATION DETAILS

For studying polymer melts of semiflexible chains in bulk, in confinement and with free surface, a new coarse-grained model (a new variant of bead-spring model) was developed recently [S1]. A short-range attractive potential between non-bonded monomer pairs is added such that the pressure P can be tuned at zero. For keeping the chain conformations which only weakly depend on the temperature T, the conventional bond-bending potential [S2, S3] is replaced by a new bond-bending potential, paramterized to conserve conformational properties at $T = 1\epsilon/k_B$. This model was tested by studying a bulk polymer melt of weakly semiflexible chains under cooling. For such a system, the monomer density at pressure $P = 0.0\epsilon/\sigma^{-3}$ is $\rho = 0.85\sigma^{-3}$ and the entanglement length $N_e = 28$ monomers. Starting from a fully equilibrated polymer melt consisting of $n_c = 2000$ polymer chains of $n_m = 50$ monomers with the Kuhn length $\ell_K \approx 2.66\sigma$ [S4], molecular dynamics (MD) simulations were performed in the NPT ensemble at $P \approx 0\epsilon/\sigma^3$ and constant temperature T following a stepwise cooling strategy. The temperature was reduced in steps of $\Delta T = 0.05\epsilon/k_B$ from $T = 1.0\epsilon/k_B$ to $0.05\epsilon/k_B$ with a relaxation time between each step of $\Delta t = 60000\tau \approx 8.3\tau_R$ (chain conformations were stored every 500τ), $\tau_R = \tau_0 n_m^2 \approx 7225\tau$ being the Rouse time of relaxing the overall chains at $T = 1\epsilon/k_B$. The characteristic relaxation τ_0 is determined from the mean square displacement (MSD) of inner monomers [S4]. This resulted in a cooling rate of $\Gamma = \Delta T/\Delta t = 8.3 \times 10^{-7}\epsilon/(k_B\tau)$. Here k_B is the Boltzmann factor, σ, ϵ and $\tau = \sigma\sqrt{m/\epsilon}$ with a monomer mass of $m = 1$ are the Lennard-Jones units of length, energy, and time, respectively. Starting from the last configuration obtained from the NPT run at each T, further MD simulations choosing the time step $\delta t = 0.01\tau$ were performed in the NVT ensemble. Estimates of MSD of inner 12 monomers, $g_1(t)$, characterizing the mobility of chains are shown in Figure S1. All MD simulations are performed using the package

ESPResSo++ [S5, S6].

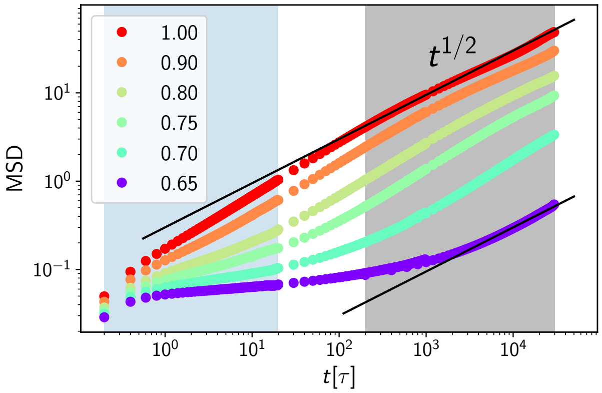

FIG. S1. Adapted from [S1]: Mean square displacements (MSD) $g_1(t)$ of inner 12 monomers as a function of time for bulk polymer melts containing $n_c = 2000$ chains of length $n_m = 50$ monomers at various selected temperatures $T[\epsilon/k_B]$, as indicated. For testing our data-driven approach, we take MD simulation trajectories at every 200τ in the time frame between 200τ and $3\times10^4\tau$ (shaded gray colored region). We have also taken the trajectories at every 0.2τ in the time frame between 0.2τ and 20τ (shaded blue colored region) for comparison. The Rouse-like scaling law $g_1(t) \sim t^{1/2}$ is represented by straight lines.

S-II. STATIC PROPERTIES OF POLYMER MELTS

The conformation of a polymer chain usually is described by the radius of gyration R_g and end-to-end distance R_e as follows,

$$R_g = \sqrt{\frac{1}{n_m}\sum_{i=1}^{n_m}(\mathbf{r}_i - \mathbf{r}_{\text{c.m}})^2} \quad \text{with} \quad \mathbf{r}_{\text{c.m.}} = \frac{1}{n_m}\sum_{i=1}^{n_m}\mathbf{r}_i \tag{S1}$$

and

$$R_e = \sqrt{(\mathbf{r}_{n_m} - \mathbf{r}_1)^2} \tag{S2}$$

where $\mathbf{r}_i$ is the coordinate of ith monomer in the chain, and $\mathbf{r}_{\text{c.m.}}$ is the center of mass of chain. As mentioned above, the profiles of probability distributions of R_g and R_e for all 2000 chains in the melt should remain the same within fluctuation at all temperatures, as shown in Figure S2.

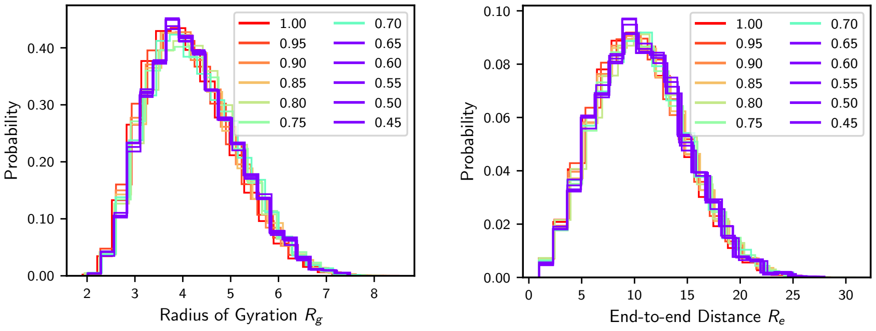

(a) (b)

FIG. S2. Probability distributions of radius of gyration (a) and end-to-end distance (b) at several selected temperatures T, as indicated.

S-III. DATA-DRIVEN APPROACH

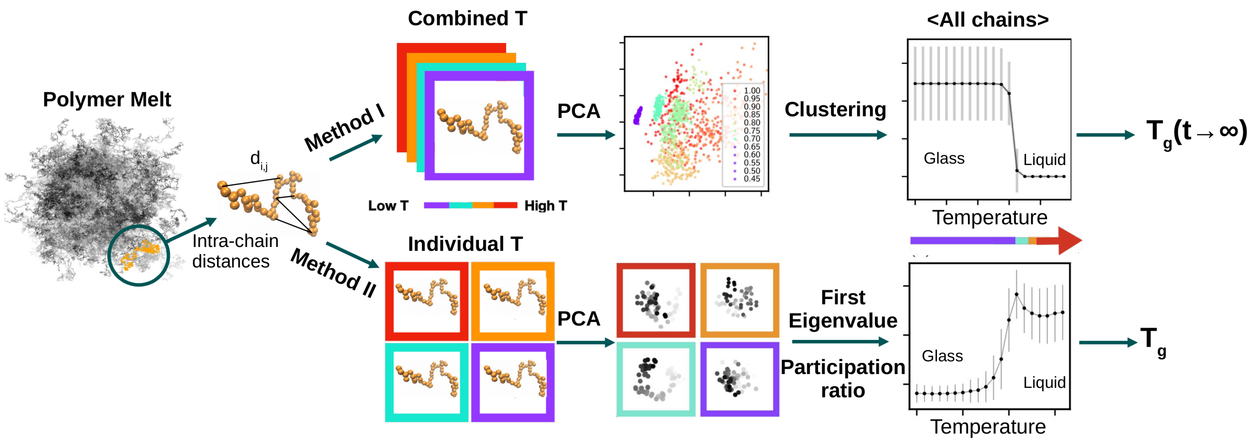

FIG. S3. Schematic representation of the two methods employed in the paper for the determination of glass transition temperature T_g. The workflows uses intra-chain distances of individual chains from the melt. It can be applied in two independent ways: by projecting with PCA combined data from temperatures followed by clustering, where the change in cluster indexes indicates T_g (Method I, upper raw). Or by applying PCA to each temperature separately and using changes in leading eigenvalues or participation ratio as the criteria to define T_g (Method II, lower raw).

S-IV. VARIANCE EXPLAINED RATIO OF PCA PROJECTIONS

Four leading PCs containing $\approx 60\%$ of a variance of the data based on the gap in the variance explained ratio[1] (Figure S4).

[1] The ratio between the variance of each principal component and the total variance.

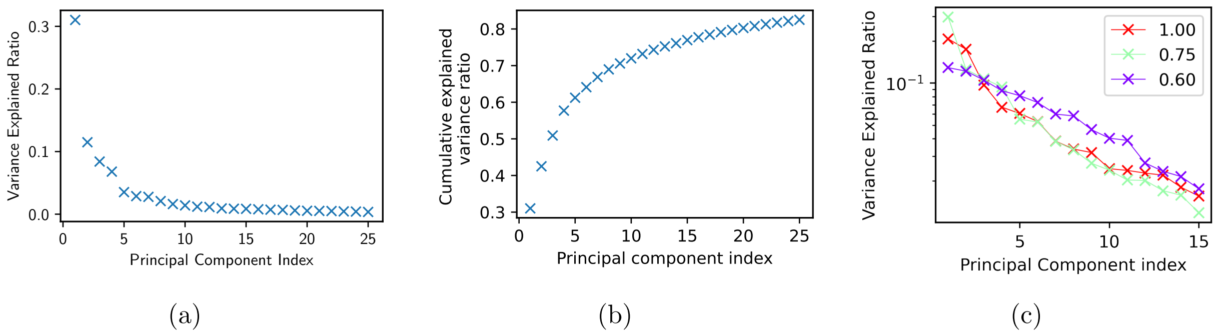

FIG. S4. (a) Variance explained ratio plotted vs. principal component index for data combined from all T (Method I). Principal components are ranked by the amount of variance they capture in the original data. The gap before the fifth component, suggesting the leading 4 components are important. (b) Cumulative summation of the explained variance (presented in (a)) shows that first four PCs describe $\approx 60\%$ of variance in the data. (c) Variance explained ratio plotted vs. principal component index for three selected temperatures $T[\epsilon/k_B] = 1.0, 0.75, 0.6$ (Method II). The decay rates of eigenvalues are different. The participation ratio reflects this decay being highest when the decay rate is slowest.

S-V. CLUSTERING METHOD – DBSCAN

The projected data is grouped using the unsupervised learning technique known as clustering analysis. Typically grouping is done based on some common property, i.e. closeness in space, similarities in densities (same number of neighbours within a cut-off separated by low-density regions), etc. The choice of clustering technique is then motivated by the properties based on which the data should be grouped, properties of the data itself (e.g. the number of data points, dimensionality etc.), as well as by some additional knowledge about the system. All clustering techniques require initial parameters to define clusters. Then they try to arrange the data points into groups. As a result each data point is assigned an index corresponding to the group it belongs to. Such an index is also called a cluster index (ID).

In our case, each data point in the projection corresponds to one configuration of a chain at a given time and temperature. Expecting that the configurations at lower temperatures will be much closer together and disordered at higher temperatures for clustering we chose the density-based spatial clustering of applications with noise (DBSCAN)[S7]. The acronym was given by the authors of the clustering algorithms (Martin Ester, Hans-Peter Kriegel, Jörg Sander and Xiaowei Xu, 1996) and for our case, it refers to distances and densities in four-dimensional projected PCA space, where now one chain is represented as a point in

four dimensions. It is designed to find high dense regions in space as separate clusters and assign all sparse points as unclustered or "noise".

DBSCAN parameters. DBSCAN groups points that are close to each other based on a distance measure (usually Euclidean distance[2]) and a minimum number of points within some cut-off.

There are two parameters one needs to specify for DBSCAN:

1) A cut-off value to define proximity in space – minimum distance (ϵ_d): indicates the radius within which points to be regarded as neighbors. Two points are regarded as neighbors if the distance between them is less than or equal to this cut-off value ϵ_d. Small ϵ_d is used for defining denser clusters. On the other hand, if ϵ_d is too high, the majority of the data will be in the same cluster. There are strategies to choose ϵ_d [S8].

2) Number of nearest neighbors (NN): number of points within distance ϵ_d to make the smallest cluster. As a rule of thumb, the number of minimum points should be greater than a dimensionality of points and typically chosen as twice the number of dimensions, but it may be necessary to choose larger values for different data sets.

DBSCAN has a number of advantages: it can find non-linearly separable clusters (clusters of any shapes); the number of clusters is not a prior parameter in the method (compared to many other methods); and most importantly for us, it has the notion of noise.

Evaluation of clustering results. To quantify the quality of the clustering results depending on the chosen parameters we used V-measure (or normalised mutual information) score [S9]. For this measure, reference cluster IDs for each point are provided. Those reference IDs are called true labels or ground truth. For our system, we define reference states (ground truth from Ref. [S1] using the volume change) as cluster IDs = -1 for the states above T_g ($T > 0.65\epsilon/k_B$), otherwise cluster IDs = 0.

The V-measure is an average of the other two measures: homogeneity and completeness. Homogeneity is defined using Shannon's entropy.

$$hom = \begin{cases} 1 & \text{if } H(C) = 0 \\ 1 - \frac{H(C|K)}{H(C)}, & \text{otherwise.} \end{cases} \tag{S3}$$

[2] For the points p and q with Cartesian coordinates p_i and q_i in $n-$dimensional Euclidean space, the distance is defined as

$$d(p,q) = \sqrt{(p_1 - q_1)^2 + (p_2 - q_2)^2 + \cdots + (p_n - q_n)^2}.$$

where C is the target clustering (ground truth), $H(C) = -\sum_{c=1}^{|C|} \frac{n_c}{M} \log \frac{n_c}{M}$, $H(C|K) = -\sum_{c=1}^{|C|} \sum_{k=1}^{|K|} \frac{n_{ck}}{M} \log \frac{n_{ck}}{n_k}$, M is the size of a data set, n_{ck} is number of samples with the cluster ID c in cluster k and n_k the total number of samples in cluster k. The homogeneity is equal to 1 when every sample in cluster k has the same cluster ID c. $0 \leq hom \leq 1$, with low values indicating a low homogeneity.

Completeness measures whether all similar points are assigned to the same cluster, it is given by

$$comp = \begin{cases} 1 & \text{if } H(K) = 0 \\ 1 - \frac{H(K|C)}{H(K)}, & \text{otherwise.} \end{cases} \tag{S4}$$

The completeness is equal to 1 when all samples of cluster ID c have been assigned to the same cluster k, $0 \leq comp \leq 1$. Normalised mutual information or V-measure is a measure of the goodness of a clustering algorithm considering the harmonic average between homogeneity and completeness. It is given by

$$\text{V}_{\text{measure}} = 2 * \frac{hom * comp}{hom + comp}. \tag{S5}$$

$0 \leq \text{V}_{\text{measure}} \leq 1$, where high values indicate good clustering.

The clustering score was computed using the python scikit-learn package [S10]. As shown

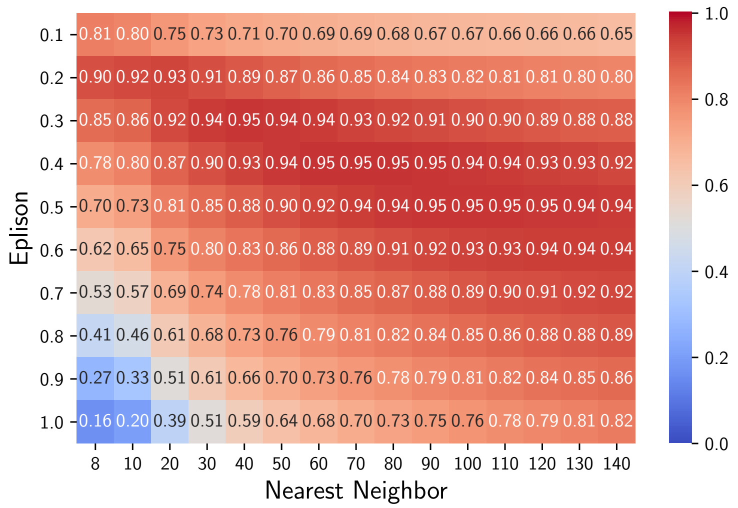

FIG. S5. Heat-map of V-measure on the PCA embedding averaged over all chains with Method I, i.e. combined temperatures using 3000 frames. The best parameter set for DBSCAN is chosen where the V-measure value is maximum.

in Figure S5, it is possible to obtain good clustering scores for quite a big range of NN and ϵ_d values on the PCA embedding. For the data shown in the main text we used the Euclidean distance ($\epsilon_d = 0.3$) to create a neighbourhood and the minimum number of points ($NN = 40$) to form a dense region.

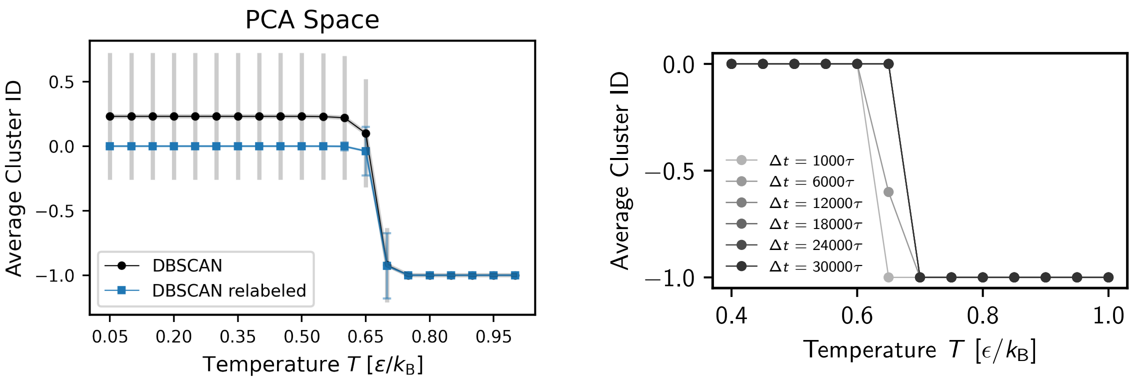

(a) (b)

FIG. S6. Average clustering results in PCA space. (a) Average clustering results by defining the error bar as deviation from the average cluster ID (black). If we relabel the clusters (blue) to make sure that there is only one cluster in the glassy state, we immediately observe that the error bars (blue bars) in the glassy state drop down to zero and they are maximum near the transition. (b) Averaging using median instead of mean for DBSCAN cluster indices (IDs). The transition at T_g becomes even sharper with this type of averaging.

Defining average cluster ID $\langle n(T) \rangle$. We perform DBSCAN clustering on the 4-dimensional projections to PCA space for each chain separately. DBSCAN defines the high-temperature states as sparse or "noise" (and assigns them with cluster ID = -1) and the low-temperature glassy state as a cluster(s) (Cluster IDs $\geq$ 0). The change in cluster indices after $T = 0.65\epsilon/k_B$ is prominent (Figure 1d). Then we repeat this clustering on each chain present in the system (2000 chains), meaning each chain at each frame and temperature will get a cluster ID value (in our example for PCA space cluster IDs are -1, 0, 1 or 2). We observe that for some of the projections in the glassy state, there is more than one group/cluster found corresponding to cluster IDs 0, 1, 2. Then we average over all cluster IDs for all chains $\langle n(T) \rangle$ that were simulated at each temperature. If we use the mean value for averaging, the average cluster IDs are higher than zero and reflect that more than one cluster was found for this temperature. Such deviation in cluster IDs is shown in large constant error bars in Figure S6a (black line). If we assume that there can be only one cluster in a glassy state and combine all the cluster IDs with 0, 1 or 2, the error bars in

the glassy state drop down to zero and they are maximum near the transition region, where assigning the points to a group is the most challenging (Figure S6a (blue line)). If we use the median for averaging we do not have such deviations from zero (Figure S6b).

S-VI. PCA ON COMBINED CHAINS

In the main text, we perform PCA on intra-chain distances for an individual chain over simulation frames, followed by taking an averaging over all chains presented in the system. Here we perform PCA on all 2000 chains together. We use the input data matrix $\mathbf{X} \in \mathbb{R}^{M \times L}$, where L is the number of descriptors (e.g. internal distance of a single chain of chain length $n_m = 50$: $L = n_m \times (n_m - 1)/2 = 1225$, same as before), and M is the number of observations (e.g. number of chains multiplied by number of temperatures, $2000 \times 20 = 40000$). We observe the Gaussian-like distribution for all temperatures in Figure S7 within fluctuations.

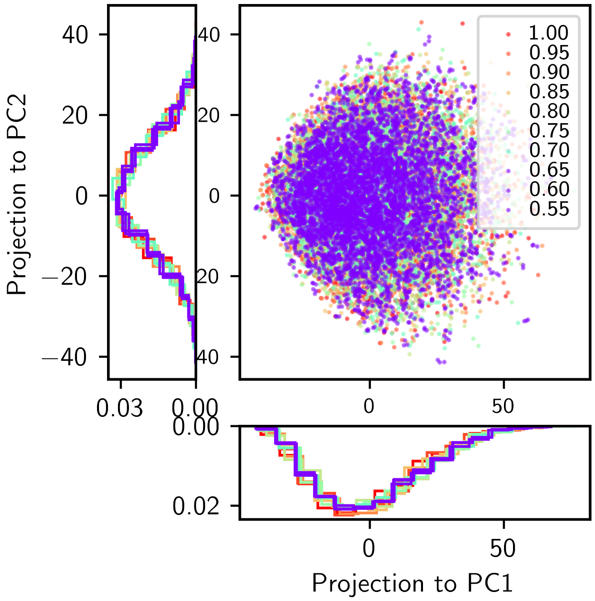

FIG. S7. PCA projections of all chains, and the distributions of PCs for several selected T, as indicated. No temperature-dependent separation in the low dimensional projections is observed.

S-VII. INTERPRETATION OF THE PROJECTION TO LEADING PRINCIPAL COMPONENTS

In order to interpret the obtained projections to PCs, we calculate the Pearson correlation coefficient [S11] between the input features (intra-chain distances in space) and the projections to leading PCs (first and second) obtained by Method I. The most correlated intra-chain distances and their locations in the chain (chemical distance along the chain) are identified for all chains and their probability distribution functions for all chains are shown in Figure S8a-c. There are no characteristic positions in a chain (e.g. end monomers) or distances which highly correlate with the projections to leading PCs. For most of the chains, the chemical distance with the highest correlations is normally distributed with a peak of around 30 monomers. Moreover, some intermediate distances, but not the longest, are dominant. This can be also seen in Figure S8a,b, where intra-chain distance distributions have no peaks at higher values. We should stress once more that due to standardisation of the distances PCA accounts for relative changes rather than the absolute displacement values which suggests that the rearrangements in long, medium, and short ranges have equal importance.

Figure S8d shows averaged Pearson correlation coefficients between the projections to the leading PCs, a radius of gyration R_g (Eq. (S1)), and an end-to-end distance R_e (Eq. (S2)). Results are obtained by taking the average over all 2000 chains. We find that both R_g and R_e are correlated with the first PC.

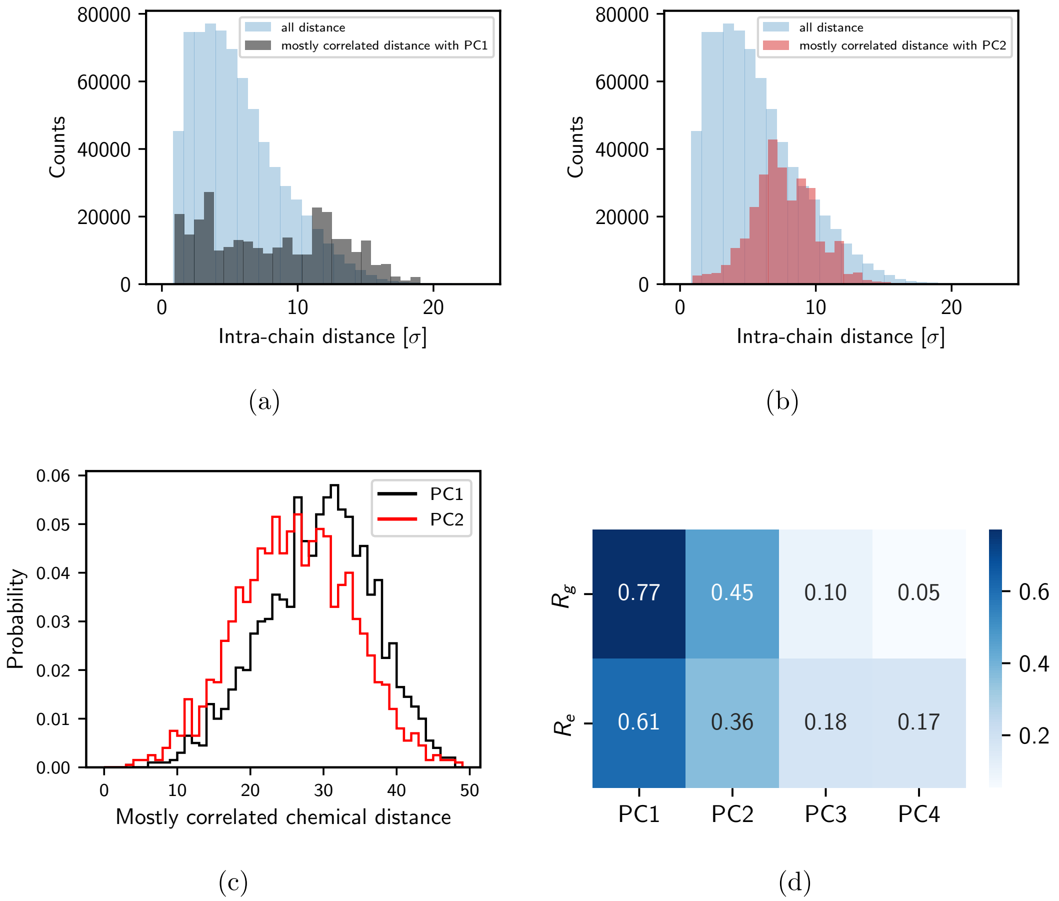

FIG. S8. Distributions of intra-chain distances mostly correlated (correlation higher than 0.9) with projections to the first (a) and second (b) principal components for all chains compared with all intra-chain distances distributions (blue bars). (c) Distribution of respective chemical distances of intra-chain distances highly correlated with the projection of two leading PCs. No preferences for longer/shorter distances ranges can be observed. d) Pearson correlation coefficients between projections to the leading PCs, a radius of gyration (R_g), and an end-to-end distance (R_e).

S-VIII. PROJECTIONS OF A CHAIN AFTER PERFORMING PCA INDEPENDENTLY AT EACH TEMPERATURE

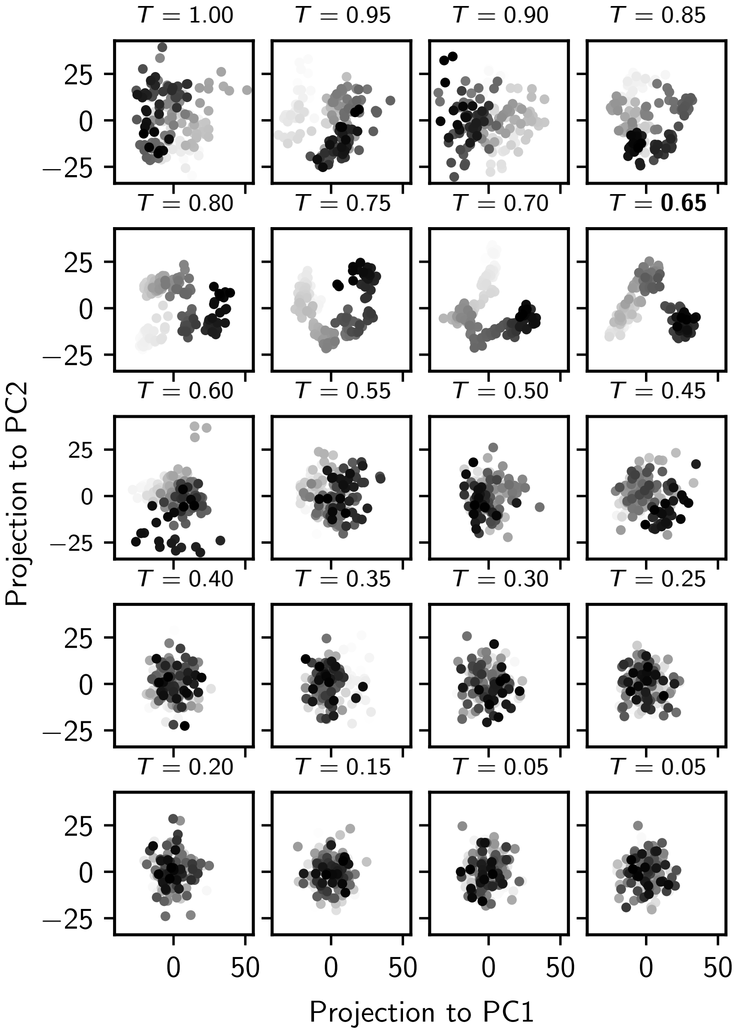

FIG. S9. Temperature dependent PCA projections for a randomly selected chain at each temperature T, as indicated. Color gradient for all projection correspond to the simulation time starting from light-gray to black. The fluctuations of data have the same magnitude after we standardise the input distances at each T. Hence, the PCA projections at $T \gg T_g$ and $T < T_g$ look visually similar. Around T_g, we see the change in the shape of the projections. This change is quantified using the first eigenvalue and the participation ratio as discussed in the main text. Data taken from the time window between 200τ and $3 \times 10^4\tau$ (gray area in Figure S1).

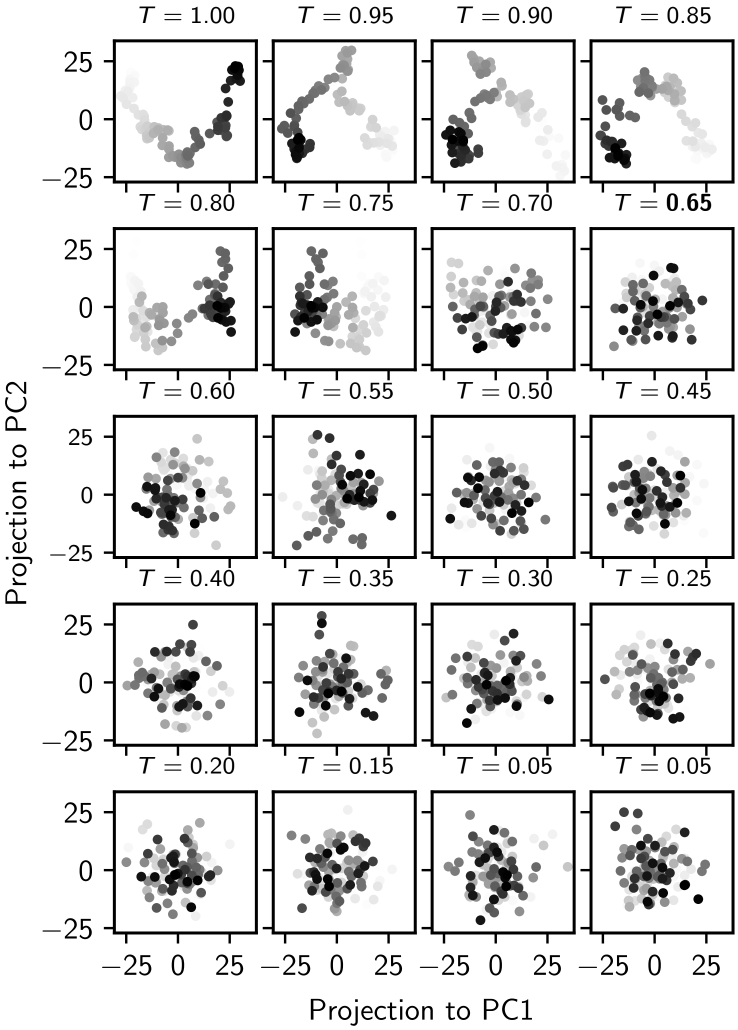

FIG. S10. Temperature dependent PCA projections for a randomly selected chain at individual T from a short MD time data (up to 20τ) (blue area in Figure S1). No sharp jump in the first eigenvalue or PR around $T_g \approx 0.65\epsilon/k_B$ is observed.

S-IX. RESULTS WITH REDUCED NUMBER OF DESCRIPTORS

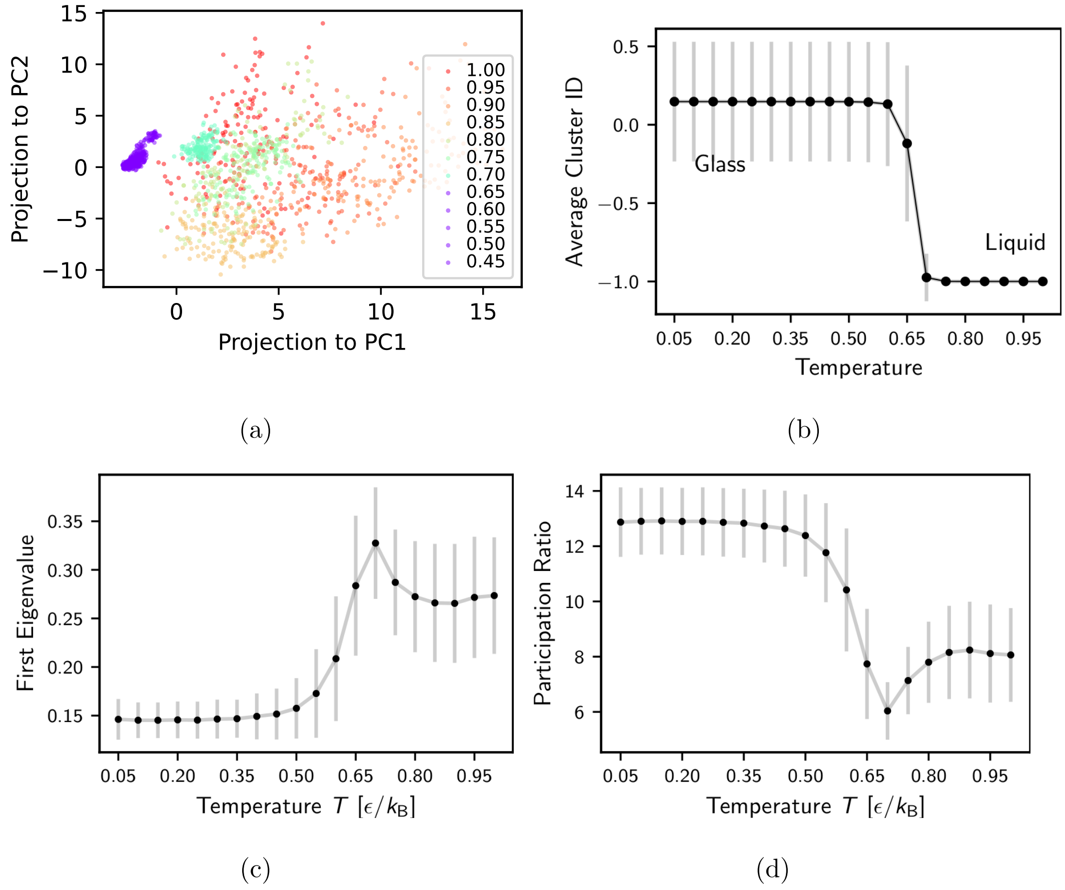

FIG. S11. The same analysis as in the main text done for reduced number of input descriptors (intra-chain distances calculated excluding four consecutive monomers). (a) PCA projection of the same selected chain as given in Figure 1b of main text. (b) Average cluster IDs $\langle n(T) \rangle$ as in Figure 1d. The first eigenvalue (c) and PR (d) from individual temperature analysis (compare to Figure 3).

In the main text, we use the input data matrix $\mathbf{X}_c \in \mathbb{R}^{M \times L}$, where L is the number of descriptors (the intra-chain monomer-monomer distances of a single chain), and M is the number of observations ($M = 3000$ for Method I and $M = 150$ for Method II). To reduce the short-range and highly correlated features we skip Δ_m consecutive monomers such that only $\lfloor \frac{n_m}{\Delta_m + 1} \rfloor$ monomers with monomer indices $i \in \{k(\Delta_m + 1) + 1 : k \text{ is an integer with } k = 0, 1, \dots, \lfloor \frac{n_m}{\Delta_m+1} \rfloor - 1\}$ from n_m monomers in each chain are selected. E.g. for $n_m = 50$ and $\Delta_m = 4$ the monomers with indices $i \in \{1, 6, ..., 41, 46\}$ are selected. Our new descriptor space ($L = 10 \times (10 - 1)/2 = 45$, M remains the same) is relatively lowedimensional

compared to the original ($L = 1225$). In Figure S11, we plot the single chain PCA projection, cluster IDs averaged over all chains, the first eigenvalue and PR from individual temperature analysis (as described in the main text) versus the temperatures T. All results are similar in nature after reducing the input feature space by excluding the contributions from the monomer pairs having chemical distances less than $(\Delta_m + 1)$ along identical chains (data taken from the gray area of Figure S1). Nonetheless, to avoid the discussion on how many monomers one can skip for each specific system and make the description of our method more general we present the data with all intra-chain distances in the main text as PCA accounts for the highly correlated distances.

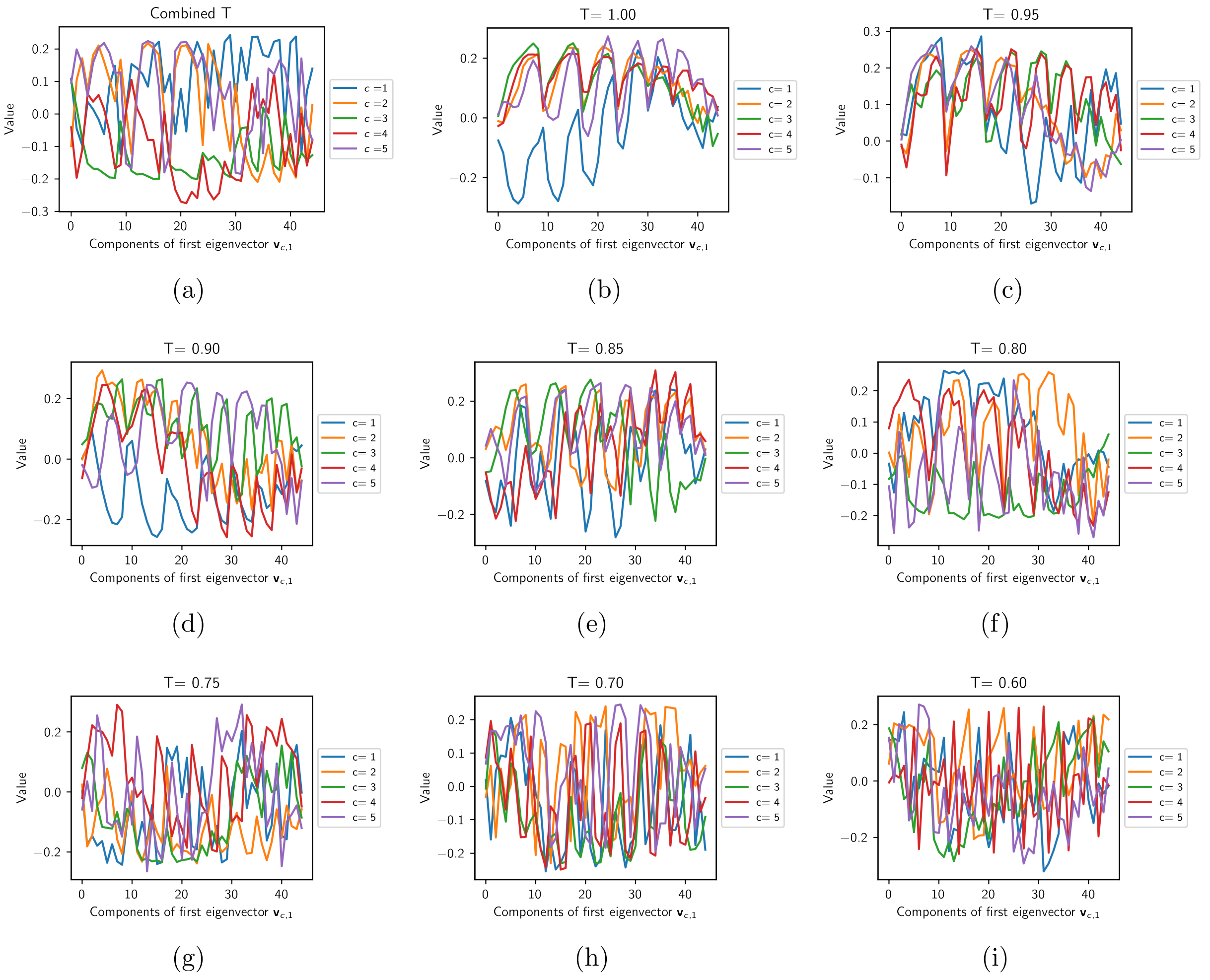

FIG. S12. First eigenvectors ($\mathbf{v}_{c,1} \in \mathbb{R}^{45}$) of PCA applied independently to four randomly selected chains (on subset of 45 internal distances): (a) for Method I (combined temperatures); (b)-(c) Method II: different selected temperatures $T[\epsilon/k_B] = 1.00, \cdots, 0.60$ respectively. The x-axis shows the index of components of the first eigenvector ($\mathbf{v}_{c,1} = [v_1 \quad v_2 \quad \cdots \quad v_{45}]$) and the y-axis shows the value of each component. The chains in all plots are the same and indexed as $c = 1, 2, 3, 4, 5$.

It is important to note the distinction between the eigenvectors' spaces used for Method I and II. In the first case we have one set of $\mathbf{v}_{c,k}$, $k = 1, ..., P$ over all temperatures. For Method II there are different independent sets of eigenvectors for each T $\mathbf{v}_{c,k}(T)$. The examples of the first eigenvectors for four different chains are shown in Figure S12 for (a) Method I (combined temperatures) (b-i) Method II (individual temperatures). For combined temperatures as well as for lower individual temperatures there are no common pattern/peaks for different chains. However, for the high temperatures (Figure S12b-e) we observe the similarity in the first eigenvectors, which suggests the importance of chemical distances at around 30 for the PCA projections obtained at this temperatures. The same importance was found for the PCA projections of the Method I (see Figure S8c) and requires further study.

[S1] H.-P. Hsu and K. Kremer, A coarse-grained polymer model for studying the glass transition, J. Chem. Phys. **150**, 091101 (2019).

[S2] R. Faller, A. Kolb, and F. Müller-Plathe, Local chain ordering in amorphous polymer melts: influence of chain stiffness, Phys. Chem. Chem. Phys. **1**, 2071 (1999).

[S3] R. Everaers, S. K. Sukumaran, G. S. Grest, C. Svaneborg, A. Sivasubramanian, and K. Kremer, Rheology and microscopic topology of entangled polymeric liquids, Science **303**, 823 (2004).

[S4] H.-P. Hsu and K. Kremer, Static and dynamic properties of large polymer melts in equilibrium, J. Chem. Phys. **144**, 154907 (2016).

[S5] J. D. Halverson, T. Brandes, O. Lenz, A. Arnold, S. Bevc, V. Starchenko, K. Kremer, T. Stuehn, and D. Reith, Espresso++: A modern multiscale simulation package for soft matter systems, Comput. Phys. Commun. **184**, 1129 (2013).

[S6] H. V. Guzman, N. Tretyakov, H. Kobayashi, A. C. Fogarty, K. Kreis, J. Krajniak, C. Junghans, K. Kremer, and T. Stuehn, Espresso++ 2.0: Advanced methods for multiscale molecular simulations, Comput. Phys. Commun. **238**, 66 (2019).

[S7] M. Ester, H.-P. Kriegel, J. Sander, and X. Xu, A density-based algorithm for discovering clusters in large spatial databases with noise, in Proceedings of the II International Conference on Knowledge Discovery and Data Mining, KDD'96 (AAAI Press, 1996) pp. 226–231.

[S8] E. Schubert, J. Sander, M. Ester, H. P. Kriegel, and X. Xu, Dbscan revisited, revisited: Why and how you should (still) use dbscan, ACM Trans. Database Syst. **42** (2017).

[S9] A. Rosenberg and J. Hirschberg, V-measure: A conditional entropy-based external cluster evaluation measure, in Proceedings of the 2007 Joint Conference on EMNLP-CoNLL (Association for Computational Linguistics, Prague, Czech Republic, 2007) pp. 410–420.

[S10] F. Pedregosa, G. Varoquaux, A. Gramfort, V. Michel, B. Thirion, O. Grisel, M. Blondel, P. Prettenhofer, R. Weiss, V. Dubourg, J. Vanderplas, A. Passos, D. Cournapeau, M. Brucher, M. Perrot, and E. Duchesnay, Scikit-learn: Machine learning in python, J.

Mach. Learn. Res. **12**, 2825–2830 (2011).

[S11] K. Pearson and F. Galton, Vii. note on regression and inheritance in the case of two parents, P. R. Soc. London **58**, 240 (1895).
